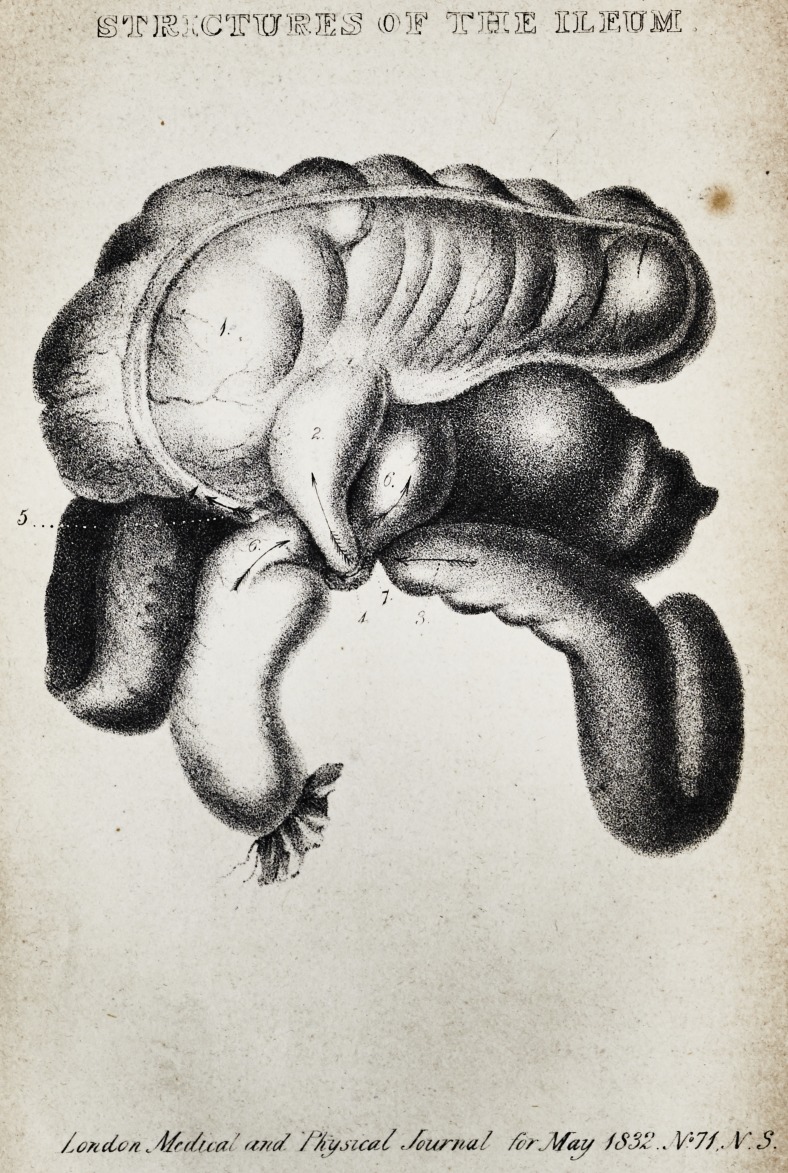# Case of Strictures of the Ileum, Attended by Fæcal Vomiting

**Published:** 1832-05

**Authors:** 

**Affiliations:** Physician to the General Dispensary, and to the Infant Orphan Asylum.


THE LONDON

### Medical and Physical Journal.

399, VOL. LXVII.] MAY 1832. [71, *New Series*.

"For many fortunate discoveries in medicine, and for the detection of numerous errors, the world is indebted to the rapid circulation of Monthly Journals; and there never existed any work to which the Faculty, in Europe and America, were under deeper obligations than to the 'Medical and Physical Journal of London,' now forming a long but invaluable series."—RUSH.

### ORIGINAL PAPERS, AND CASES

### OBTAINED FROM PUBLIC INSTITUTIONS AND OTHER AUTHENTIC SOURCES.

---

### STRICTURES OF THE ILEUM.

*Case of Strictures of the Ileum, attended by Fæcal Vomiting.* By Dr. ROBERTS, Physician to the General Dispensary, and to the Infant Orphan Asylum.

[WITH AN ENGRAVING.]

ABOUT a fortnight since, I was requested by my friend Mr. BURN, of St. Paul's Church-yard, to visit with him a patient, named James Beckley, about thirty-five years of age, and by trade a dyer, who had been attacked on the preceding Saturday (this being Monday night,) with pain in the bowels, attended by constipation. He had used the common saline aperients with calomel, but without any effect. The patient became worse on the Sunday, and, owing to Mr. Burn's absence, was visited by Mr. Holmes, of Old Fish street, who bled him, and who also prescribed some more cathartic medicine. On the morning of the day on which I first saw him, Mr. B. had placed him in a warm bath, which relieved the pain, but produced no sensible effect upon the bowels, notwithstanding he had fainted, either during the immersion or very immediately afterwards.

I found him lying on his back, with his knees bent towards the abdomen, a pallid and anxious countenance, a fluttering pulse, beating rather more than one hundred in the minute, with a red and moist tongue, great thirst, and excessive tumefaction of the bowels, and great tenderness in the whole tract of the colon, more especially about the situation of the cœcum. Eighteen leeches were applied, which bled profusely, and materially relieved the pain and tumefaction, but was not followed by any evacuation from the bowels. On

Tuesday morning, a blister was ordered, which was rendered desirable, not only on account of the intestinal obstinacy and pain, but also from there being some threatenings that the stomach was about to become irritable. The pain was again somewhat mitigated by this, but it was not followed by the slightest relief from the bowels. This state of things continued with but little or but very slight alteration for four or five days, the bowels remaining obstinate, in spite of the exhibition of croton oil and turpentine, together with other cathartics. The only material change which was observed from the application of the leeches and the blister, was the pulse becoming "*plus prononcée*," but unaltered in velocity.

He now began to complain seriously of the distention of the belly, which was so considerable as to prevent his drinking with any thing like comfort to himself. At length the stomach became exceedingly irritable, and he commenced vomiting, first a darkish green-coloured bile, and afterwards feculent matter. Opium alone, and calomel and opium, were now given in tolerably large doses; and enemata, consisting of infusion of senna, castor oil, croton oil and aloes, and turpentine, were from time to time thrown up, by means of Maw's injecting syringe, but without any avail. Frictions also of camphor liniment, containing two drops of croton oil to the half-ounce, were assiduously rubbed over the abdomen. The vomiting now ceased, having continued more or less for about three days. The urine, during the whole of the continuance of the disease, was healthy in appearance and quantity, but, after taking the spirits of turpentine, (which he did about the third day after I commenced attending him,) it became strongly impregnated with the peculiar terebinthinous smell, which it continued to retain until he died, a period of full ten, if not twelve, days.

He remained nearly in the same condition for four or five days longer, the pain neither increasing nor diminishing, and the constipation being as determined as ever. The stomach, however, now again gave way, and he recommenced vomiting of feculent matter, which continued at intervals until he died, which took place exactly at the end of a fortnight from my having first seen him.

Mr. Burn more than once introduced a candle up the rectum, which went up without obstruction to a very considerable distance, and was withdrawn without being in the slightest degree soiled. Fomentations were also assiduously employed during the whole period of his illness; and the only reason for our not repeating the warm bath was, first, the difficulty of moving him, and secondly, the fear of again producing fainting, from which, in the first instance, he was so long in being recovered.

Soon after using one of the injections, a small piece of feculent matter was passed, having nearly the following size and shape, and which confirmed us in our opinion of there being internal mechanical obstruction.

Towards the close of his life, his tongue became dry and harsh, but continued red, and there were apthæ.

The largest quantity of feculent matter thrown up during twelve hours, was about five quarts.

Permission being given to open the body, this was carried into effect about forty-eight hours after death. The examination was performed by my friends Mr. Jonathan Pereira and his brother; to the former of whom I am indebted for the account of the appearances on dissection.

The abdomen appeared much distended and very slightly discoloured, and, on moving the body in the most trifling degree, a great abundance of fæcal matter flowed from the mouth and nostrils.

On opening the abdominal cavity, the small intestines presented themselves enormously distended with wind, to nearly or quite four times their natural size. The colon, being suspected to be the seat of the mischief, was very carefully examined: the ascending portion, as well as the tranverse, were found to be natural; but the descending, or sigmoid flexure, was very much contracted, and contained a cheesy or curdly looking matter, having neither the colour nor smell of fæces.

The site of the disease was now ascertained to be two strictures of the ileum: of these, the principal and fatal one was that which enveloped this gut at its termination in the cœcum. Both of these were created by the appendix vermiformis twisting round the ileum, and afterwards forming an adhesion to another portion of the same intestine.

The adhesion of the apex of the appendix vermiformis to the ileum was of very long standing, and had the appearance of having been congenital; and until the parts were removed, and distended with air, there seemed to be a communication between the appendix and the ileum. At this part the ileum had very much the resemblance of dividing into two portions; one of which, however, on further examination, proved to be the appendix itself.

The sides of the slightest stricture were formed principally by the appendix, but partly also by that portion of the ileum which enters into or terminates in the cœcum.

The sides of the second and principal stricture were formed by the appendix and that portion of the ileum which was already strangulated by the first and slightest stricture. This stricture was so firm as not to allow of even air passing through that portion of the gut confined within it.

It is almost unnecessary to say, that the intestines at these parts were highly congested, and of a bluish-green colour.

The accompanying drawing, taken by an artist at the time, it is hoped will render the above description more intelligible.

On finding what was the real cause of the obstruction, Mr. Burn and myself questioned the wife as to his usual state of health, previous to this last and fatal attack: she said he had always been subject to costive bowels; and that, after working at the calender, or pressing of goods, both of which portions of his business he was at times called upon to perform, he frequently complained of violent pain in the spot at which the disease was situated, and that it was commonly relieved by his swallowing some stimulant, as brandy; and that he had also, from time to time, complained of feeling as if one portion of his bowels were confining, or, as she expressed it, "tying up" another; she further stated that he was in every respect a very sober and steady man.

This is one of those unfortunate cases which require no comment, the dissection shewing that no medical assistance could have possibly proved serviceable. We have no means whatever for accounting in any way for the peculiarly strangulated condition of the ileum; for it would not appear, from all we could obtain of the previous history of the case, either that he had been called upon to use any sudden or violent exertion, or that he had even been employed in either pressing or calendering for some time immediately preceding this attack. We could not find any person who had known him for a longer period than about twenty years; and these all stated, that he had always complained of this pain in the abdomen, and of the great difficulty which he had in keeping up a proper evacuation from the bowels. It would appear from this, that the disease had at least been in existence for some considerable period of time, and that it had been gradually p. 355.

STRICTURES OF THE ILEUM.

London Medical and Physical Journal for May 1832. No. 71. N. S.

on the increase; but we had not any clue whatever by which we could be enabled to either ascertain its first cause or from which we might date its commencement.

*Bridge street; March* 1*st*, 1832.

### *Explanation of the Plate.*

1. Cœcum.
2. Ileum terminating in the Cœcum.
3. Ileum continuous with the preceding portion.
4. Appendix Vermiformis, forming a stricture around the portions of the Ileum marked 2 and 3.
5. Appendix attached to the Cœcum.

6 and 6. A portion of the Ileum passing behind that portion which is marked 2: it is strictured between the appendix and the Ileum marked 2, and forms the posterior portion of the stricture which is formed around the portion of the Ileum marked 2 and 3.

7. Attachment of the Appendix to the Ileum marked 6.

## Figures and Tables

**Figure f1:**
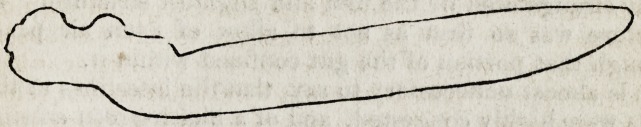


**Figure f2:**